# Neural processing of sad and happy autobiographical memories in women with depression and borderline personality disorder

**DOI:** 10.1038/s41598-024-81840-x

**Published:** 2024-12-28

**Authors:** Maria Kulesza, Katarzyna Rękawek, Paweł Holas, Dorota Żołnierczyk-Zreda, Marlena Sokół-Szawłowska, Anna Poleszczyk, Artur Marchewka, Marek Wypych

**Affiliations:** 1 Laboratory of Brain Imaging, Nencki Institute of Experimental Biology, Pasteura 3, Warsaw, 02-093 Poland; 2 Faculty of Psychology, University of Warsaw, Stawki 5/7, Warsaw, 00-183 Poland; 3 Laboratory of Psychology and Sociology, Central Institute for Labour Protection – National Research Institute, Czerniakowska 16, Warsaw, 00-701 Poland; 4 Outpatient Clinic, Institute of Psychiatry and Neurology, Sobieskiego 9, Warsaw, 02-957 Poland; 5 Department of Clinical Neurophysiology, Institute of Psychiatry and Neurology, Sobieskiego 9, Warsaw, 02-957 Poland

**Keywords:** Long-term memory, Personality, Human behaviour

## Abstract

Patients with major depressive disorder (MDD) and borderline personality disorder (BPD) are reported to have disrupted autobiographical memory (AM). Using functional magnetic resonance imaging we investigated behavioral and neural processing of the recall of emotional (sad and happy) memories in 30 MDD, 18 BPD, and 34 healthy control (HC) unmedicated women. The behavioral results showed that the MDD group experienced more sadness than the HC after the sad recall, while BPD participants experienced less happiness than HC after the happy recall. The fMRI results for sad AMs, compared to happy AMs, elicited greater activation in multiple brain regions (i.e., medial prefrontal cortex, posterior cingulate cortex, insula) linked to self-related information, emotional processing, and semantic recollection across all groups. Functional connectivity analysis revealed a significant main effect of group between the occipital cortex and precuneus and between occipital cortex and posterior cingulate cortex. The effect was driven by stronger connectivity between the occipital cortex and precuneus in the clinical groups taken together than in the HC. Our results suggest a need for stronger coordination between visual imagery and contextual recall for vivid memory retrieval in these clinical groups.

**Supplementary Information:**

The online version contains supplementary material available at 10.1038/s41598-024-81840-x.

## Introduction

The birthday gifts you received last year; your wedding day; first broken bone - the experiences we lived through are stored in the autobiographical memory (AM) system. A person is able to re-live a memory because of the numerous sensory, emotional, and contextual details that it contains. The AM plays a significant role in daily functioning. It impacts the creation of an integrated narrative of one’s life and one’s identity, problem-solving, learning, and social interactions^1–3^. However, in some psychiatric disorders (such as depression and borderline personality disorder), its functioning is disrupted.

Advancements in neuroimaging studies in healthy populations have significantly contributed to our understanding of the core brain regions involved in the retrieval of autobiographical memories (AMs), often referred to as the AM retrieval network. This network is generally understood to engage regions within the prefrontal cortex [PFC; including the anterior cingulate cortex (ACC) and the medial part of PFC (mPFC)], temporal cortex (hippocampus, amygdala - which are involved in various stages of AM retrieval and emotional processing of the memories), parietal cortex (including precuneus and angular gyrus - which are engaged in processing of self-related information, recall of specific details, and perspective taking, among other functions), and occipital cortex (engaged in, for example, visual imagery during recall:^4–6^. One of the most commonly reported regions is the mPFC, which takes part in processing self-referential information, such as during self-reflection or own traits assessment^7,8^. Connectivity studies have shown that the mPFC is functionally and structurally linked to the posterior cingulate cortex (PCC), which is involved in processing self-referential information, emotions, and episodic memory^9,10^. Some studies also discovered enhanced functional connectivity between the amygdala and hippocampus at the time of retrieval^11,12^. This implies that the recollection of emotions, handled by the amygdala, might amplify the memory search operations carried out by the hippocampus^13,14^.

AM often contains powerful emotional content. The amygdala, a region central to emotional processing, may be more engaged in recalling events that are rated as highly emotional^11^. However, some studies failed to find this relationship [e.g.,^15^]. Other investigations into the neural underpinnings of emotional AMs demonstrate varying activations depending on the valence of the memories. For instance, positive AMs have been found to elicit higher activity in regions including the mPFC, orbitofrontal cortex, temporal pole, and medial temporal cortex, whereas negative AMs elicit higher activity in the middle temporal gyrus^16^. A review by Suardi et al.^17^ included results of various studies comparing happy memories to those containing other specific emotions, like sadness or disgust. Negative memories seem to activate more varied regions depending on the specific study: middle temporal gyrus^16^, lateral orbitofrontal cortex and ventrolateral prefrontal cortex^18,19^, lateral temporal cortex^18^, and pons^19^. Authors of the review suggested that the retrieval of happy memories consistently activated regions including the ACC, mPFC, temporal gyrus, temporal pole, insular cortex, orbitofrontal cortex, hippocampus, and amygdala^16^. The authors also suggested that the heightened activity in limbic regions such as the insula and amygdala during the retrieval of happy memories may indicate a stronger experience of feelings and possibly pleasure.

Major depressive disorder (MDD) and borderline personality disorder (BPD) are distinct disorders; however, they share several commonalities in the processing of emotional information, including emotional dysregulation and cognitive distortions, both of which are related to the recall of personal past events. MDD, as defined by the Diagnostic and Statistical Manual of Mental Disorders, Fifth Edition (DSM-5), is characterized by persistent low mood, loss of interest and/or pleasure in most activities, sleep disturbances, fatigue, feelings of worthlessness or excessive guilt, and recurrent thoughts of death or suicide, lasting for at least two weeks^20^. BPD is defined by the DSM-5 as a personality disorder characterized by pervasive instability in moods, self-image, and interpersonal relationships. Key symptoms include intense fear of abandonment, chronic feelings of emptiness, impulsive behaviors, and recurrent self-harm or suicidal behavior^20^. The existing literature suggests that in both disorders the AM recall is disturbed, however, the knowledge is currently somewhat limited. On the behavioral level, the available literature suggests, for example, that patients (either with MDD or BPD) may have an overgeneral AM recall (e.g., in MDD:^21^; in BPD:^22^), which is a tendency to recall more repeated, general events and fewer specific, particular events^22,24–26^. People with either MDD or BPD may also have greater ease in recalling negative than positive events (in depression:^27,28^; in BPD:^29–32^).

Functional magnetic resonance imaging (fMRI) investigations of AM recall in MDD have revealed disturbances in this process, however, the findings were inconsistent (likely due to the heterogeneity in study designs, experimental protocols, and the number of participants taking medication;^33–35^). For instance, a study conducted by Young et al.^21^ contrasted AM recall in MDD with a healthy control group, irrespective of the memories’ valence or specificity. The results indicated decreased activation in multiple brain areas including dorsolateral PFC, ACC, anterior and posterior insulae, hippocampus, parahippocampal gyrus, and the middle temporal gyrus in the depressed individuals. The authors suggested that the differences in the activity of the hippocampus and parahippocampal gyrus may have reflected different levels of the vividness of AMs, as both regions have been implicated in the retrieval of memory details.

Only one of the studies mentioned above looked into functional connectivity. It demonstrated that for positive AMs, the MDD group exhibited decreased amygdala connectivity with dorsal ACC and PCC, and increased amygdala connectivity with mPFC and superior temporal gyrus^35^. For the negative AMs, the MDD group showed increased amygdala connectivity with dorsal ACC, superior temporal gyrus, insula, dorsolateral PFC, PCC, thalamus, medial temporal gyrus, precuneus, and the amygdala itself. In the view of the authors, reduced coactivation between the amygdala and other regions during positive retrieval upholds abnormal processing of positive information and might be a marker for depression. It further suggests that positive AMs may not be relevant to the self for individuals with MDD, as there was lower connectivity of the amygdala with regions processing the self. Conversely, increased amygdala connectivity for negative AMs indicates their prominence and significance, and more straightforward recall.

In comparison, investigations into the neural mechanisms of AM recall in BPD are less extensive and none studied functional connectivity. Two of the studies using fMRI found differences in brain activation patterns when recalling negative unresolved and resolved memories^36,37^. Unresolved memories—those perceived as highly significant, emotionally charged, and difficult to cope with—were contrasted with resolved memories, which are viewed as overcome, integrated, and less emotionally intense. For instance, a study by Beblo et al.^37^ observed that, in the BPD group, unresolved memories activated the insula, amygdala, ACC, PCC, and occipital cortex more than resolved memories did. The authors suggested this might reflect the heightened emotional experiences during unresolved recall and greater effort to control these emotional responses.

When comparing the neural mechanisms underlying AM recall in both disorders, it is notable that both MDD and BPD groups show higher activation of the ACC^34,36^, amygdala^33,35,37^, hippocampus^33,34^, precuneus, and cuneus^35,37^ in response to negative (in MDD studies) and to resolved/unresolved negative (in BPD studies) memories. This seemingly shared pattern of activation suggests that there might be similarities in the alterations present during AM recall in these clinical groups. Additionally, when looking at previous works on processing of negative stimuli in general, MDD and BPD patients may have similar dysfunctional activation of the prefrontal cortex and hyperactivation within the amygdala^38,39^. Moreover, some of the symptomatology of the disorders overlaps and, additionally, these disorders often co-occur with each other. They share a dominant negative affect, suicidal thoughts, plans, and attempts, and a tendency to focus on negative stimuli. However, a meta-analysis by Schulze et al.^40^ that compared studies on affect processing in BPD, MDD, and healthy control groups revealed that BPD patients had higher activation of the amygdala, hippocampus, angular gyrus, and inferior frontal gyrus than groups with depression, which could be related to higher emotional reactivity in BPD and poses a question if reactivity plays a bigger role in AM processing in this population. Despite their frequent co-occurrence and similarities, it is difficult to find neuroimaging literature that compares these disorders, and even in the aforementioned meta-analysis, no studies compared MDD and BPD directly.

This study aimed to investigate how emotionally valenced (sad and happy) memories are processed in women with MDD and BPD, on behavioral and neural levels. As negative information related to self is more likely to elicit negative emotions in MDD and BPD groups, and positive information may not be experienced as salient, we expected that both groups would rate their emotional state as sadder during the task after sad AMs than the control group, and as less happy after happy AMs. To the best of our knowledge, no available studies compared MDD and BPD groups during AM recall. Therefore, we decided to run exploratory analyses whether the groups would differ in terms of their emotional state and the vividness of recall after sad and happy memories. Previous research has demonstrated that individuals with MDD and BPD experience more intense emotional reactions when recalling emotional autobiographical memories compared to healthy controls. Additionally, it has been previously established that strong emotions are frequently associated with a higher level of vividness during memory recall. Therefore, on the neural level, we predicted that for sad memories, both clinical groups would show greater activation of the amygdala, insula, ACC, the occipital cortex, and precuneus (regions processing emotional content and visual imagery of the AMs) than the HC. We also expected that the results of the BPD group would show greater activation of these regions than the MDD group during sad recall. The differences in the happy memories between the groups were left for exploration, as were the functional connectivity analyses.

## Results

Thirty unmedicated women with MDD, 18 with BPD, and 34 healthy controls (HC) took part in the study. All participants provided 5 sad (SAD) and 5 happy (HAPPY) memories a few days before the main study. During the fMRI experiment participants were asked to recall their memories and rate their emotional state and vividness of the memories. A control condition, designed as a break from emotional content, included recall of neutral, daily routines (see ‘Methods’ section for details of the demographics and the procedures).

### Behavioral results

The analysis of behavioral question about the emotional state during memory recall revealed a significant main effect of group (*F*(2, 79) = 5.14, *p* = 0.01, η_*p*_^2^ = 0.12) and a significant main effect of condition (*F*(1, 79) = 335.99, *p* < 0.001, η_*p*_^2^ = 0.81) but no significant interaction (*F*(2, 79) = 1.08, *p* = 0.3, η_*p*_^2^ = 0.03). Post hoc tests revealed that the BPD group rated their emotional state as generally sadder than the HC group (*T* = -3.19, *p* = 0.01), however, there were no significant differences between MDD and BPD (*T* = 1.76, *p* = 0.2) nor between MDD and HC groups (*T* = -1.61, *p* = 0.2). The post hoc test for the condition effect showed that during sad memories emotional state was rated as sadder in comparison to happy memories (*T* = -17.69, *p* < 0.001).

**Fig. 1:**
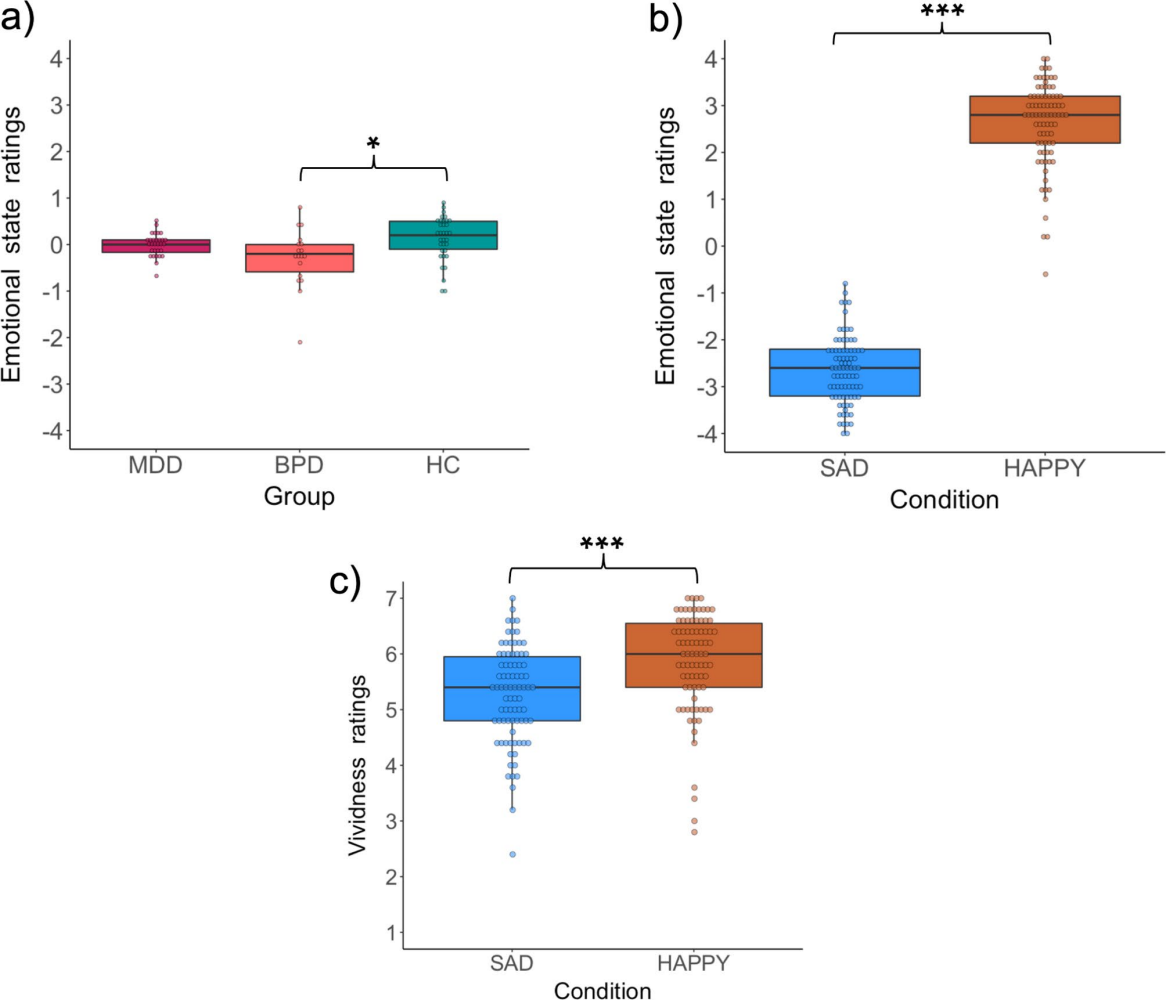
Ratings of the emotional state (**a**-**b**) and vividness (**c**) during recall. (**a**) The main effect of group. (**b**) The main effect of condition. (**c**) The main effect of the condition for the vividness question. The rating scale was changed from 1–9 points to -4-4 for visualization purposes. Dots represent individual participants. The lower and upper borders of the box correspond to the first and third quartiles, respectively. The lower and upper whiskers represent the smallest and largest data points, respectively, no further than 1.5 x interquartile range from the borders. *p < 0.05, ***p < 0.001.

The analysis of behavioral question about the vividness of recalled memories revealed a significant effect of condition (*F*(1, 79) = 56.97, *p* < 0.001, η_*p*_^2^ = 0.42), which in a post hoc test showed that sad memories were rated as less vivid than happy memories (*T* = -7.357, *p* < 0.001). The main effect of group (*F*(2, 79) = 0.6, *p* = 0.57, η_*p*_^2^ = 0.01) and interaction (*F*(2, 79) = 2.55, *p* = 0.08, η_*p*_^2^ = 0.06) were not statistically significant. The results of both behavioral questions are presented in Fig. 1.

## fMRI results

### Whole-brain GLM results

**Fig. 2:**
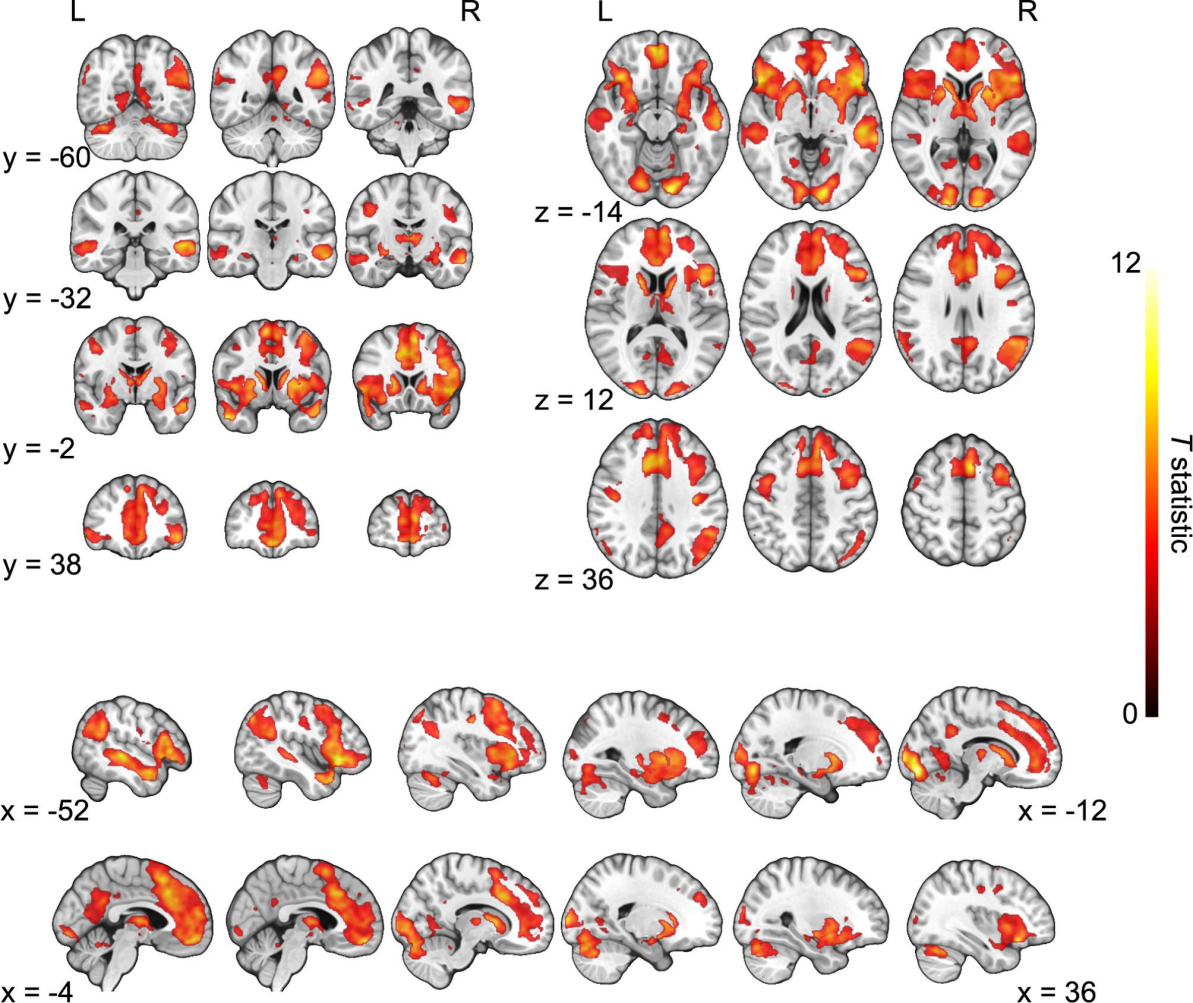
Whole-brain statistical parametric maps representing brain activation for the main effect of task (SAD + HAPPY). The results were thresholded at a voxel-wise height threshold of *p* < 0.001 (uncorrected) combined with a cluster-level extent-threshold of *p* < 0.05 and cluster size of *k* = 274 voxels and corrected with FWE rate.

The main effect of task (SAD + HAPPY, all participants taken together) revealed multiple activations (Table 1; Fig. 2) including regions within the frontal, temporal, occipital, and parietal cortices, and all the regions selected for the ROI analyses.

**Table 1:** Brain activations for the main effect of task (SAD + HAPPY).

				MNI coordinates			
Brain region	Hemisphere	Cluster size	*F*-value	x	y	z	*p* FWE peak-level
***Main effect of AM recall (SAD + HAPPY)***							
Lingual gyrus	L	7419	11.59	-1	-88	-1	**< 0.001**
Cuneus	R		9.50	16	-100	6	**< 0.001**
Middle occipital gyrus	L		9.31	-12	-102	4	**< 0.001**
Inferior orbitofrontal gyrus	L	29,138	10.64	-46	22	-6	**< 0.001**
Middle temporal pole	R		10.40	50	10	-34	**< 0.001**
Inferior orbitofrontal gyrus	R		9.82	52	22	-4	**< 0.001**
Postcentral gyrus	L	274	7.75	-38	-16	38	**< 0.001**
Precentral gyrus	R	650	6.64	42	-14	36	**< 0.001**
Supramarginal gyrus	R	398	6.00	64	-48	26	**0.002**
Angular gyrus	R		5.18	60	-56	26	**0.03**

Significant *p* values are written in bold. The results were thresholded at a voxel-wise height threshold of *p* < 0.001 (uncorrected) combined with a cluster-level extent-threshold of *p* < 0.05 and cluster size of k = 274 voxels and corrected with FWE rate. Table shows 3 local maxima of each cluster separated by a minimum of 8 mm. MNI - Montreal Neurological Institute.

**Fig. 3:**
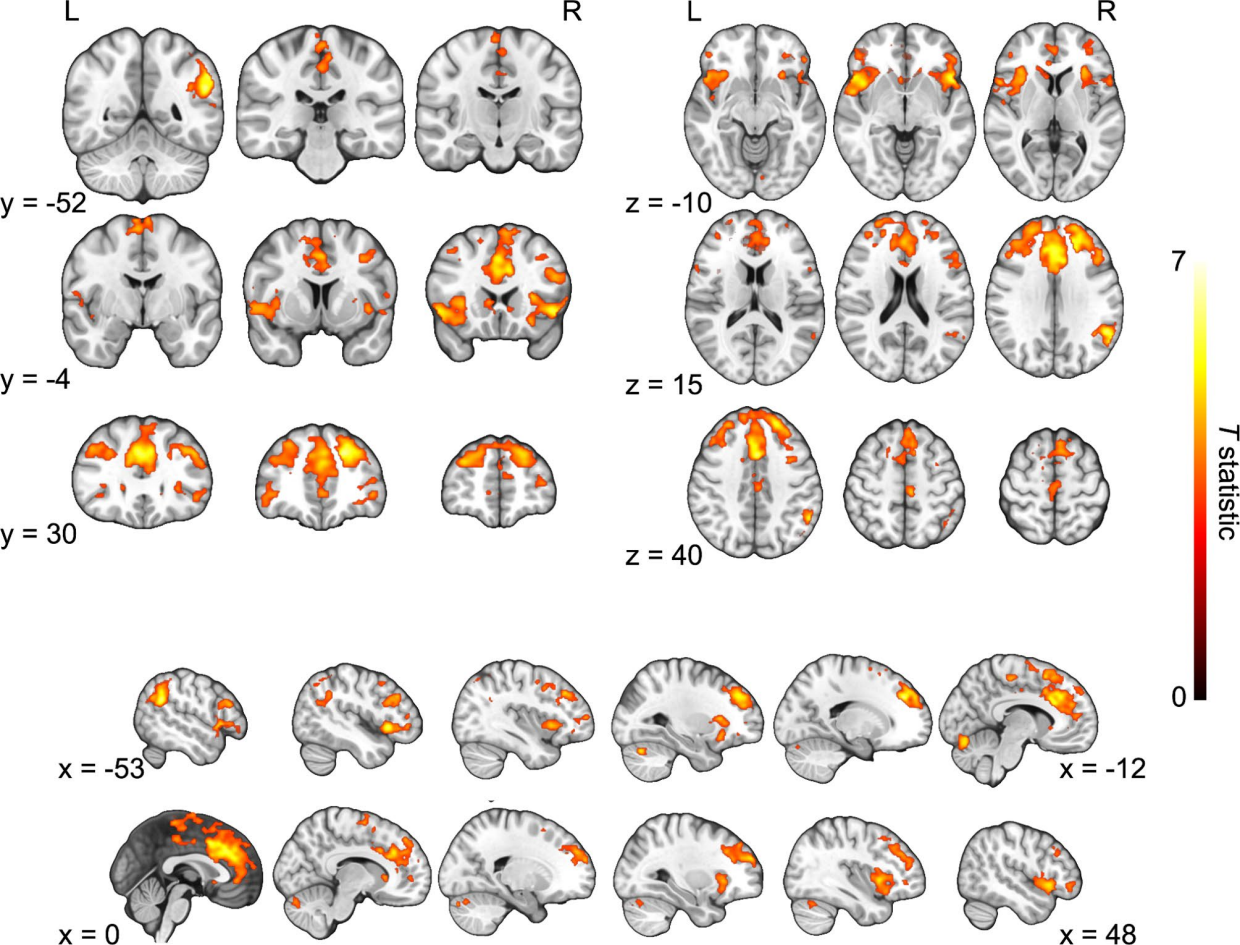
Differences in brain activation between conditions (SAD > HAPPY) for all participants. The results were thresholded at a voxel-wise height threshold of *p* < 0.001 (uncorrected) combined with a cluster-level extent-threshold of *p* < 0.05 and cluster size of *k* = 185 voxels and corrected with FWE rate.

Comparisons of sad and happy AMs across all participants (SAD > HAPPY and HAPPY > SAD) revealed that sad AMs were related to greater significant activations among multiple brain regions, including the left mPFC and dmPFC, angular gyrus, PCC, and right insular cortex (Table 2; Fig. 3). There were no significant differences in the comparison of happy AMs to sad ones.

**Table 2:** Differences in brain activation between conditions for all participants (SAD > HAPPY and HAPPY > SAD contrasts).

				MNI coordinates			
Brain region	Hemisphere	Cluster size	*T*-value	x	y	z	*p* FWE peak-level
***SAD > HAPPY***							
Superior frontal gyrus	L	10,360	6.49	-20	40	34	**< 0.001**
Middle cingulate cortex	L		6.31	-2	12	40	**0.001**
Inferior orbitofrontal gyrus	L		6.01	-50	18	-4	**0.002**
Supramarginal gyrus	L	828	6.48	-54	-52	28	**< 0.001**
Inferior parietal gyrus	L		5.94	-56	-52	38	**0.002**
Angular gyrus	L		5.41	-58	-58	32	**0.02**
Inferior frontal gyrus	R	1679	5.41	54	18	-4	**0.02**
Insular cortex	R		5.37	44	14	-6	**0.02**
Cerebellum	L	305	5.25	-28	-70	-28	**0.03**
Cerebellum	L		5.20	-6	-78	-22	**0.03**
Cerebellum	R	303	4.57	20	-84	-28	0.23
***HAPPY > SAD***							
No suprathreshold clusters							

Significant *p* values are written in bold. The results were thresholded at a voxel-wise height threshold of *p* < 0.001 (uncorrected) combined with a cluster-level extent-threshold of *p* < 0.05 and cluster size of k = 185 voxels and corrected with FWE rate. Table shows 3 local maxima of each cluster separated by a minimum of 8 mm. MNI - Montreal Neurological Institute.

The analysis of the main effect of group and interaction revealed no significant results. The additional analysis with the covariate marking current depressive episode did not yield significant results.

### ROI results

Regions of interest (ROIs) were specified a priori, as spheres with a 12 mm radius. They corresponded to the main brain regions implicated in AM recall based on previous meta-analyses and AM literature [e.g., ^4–6,45,46^]. These ROIs were: vmPFC, hippocampus, amygdala, occipital cortex, precuneus, posterior and anterior cingulate cortices, insular cortex, and angular gyrus (see the ‘Methods’ section).

The ROI analysis of the main effect of group and the group by condition interaction did not reveal significant results.

### Functional connectivity results

The same ROIs were used for the functional connectivity analysis, with a 6 mm radius. Four clusters of those regions were revealed in the hierarchical clustering procedure, performed on data taken from all participants (Table 3; see “Methods” for explanation of the procedure).

**Table 3:** Summary of clusters revealed by hierarchical clustering procedure.

Cluster name	ROIs
Cluster A	left PCC, left precuneus
Cluster B	left and right amygdalae, left and right hippocampi
Cluster C	right vmPFC, left and right AG, left and right insulae, left ACC
Cluster D	left and right occipital cortices

ROIs - regions of interest; PCC - posterior cingulate cortex; vmPFC - ventromedial prefrontal cortex; AG - angular gyrus; ACC - anterior cingulate cortex.

**Fig. 4:**
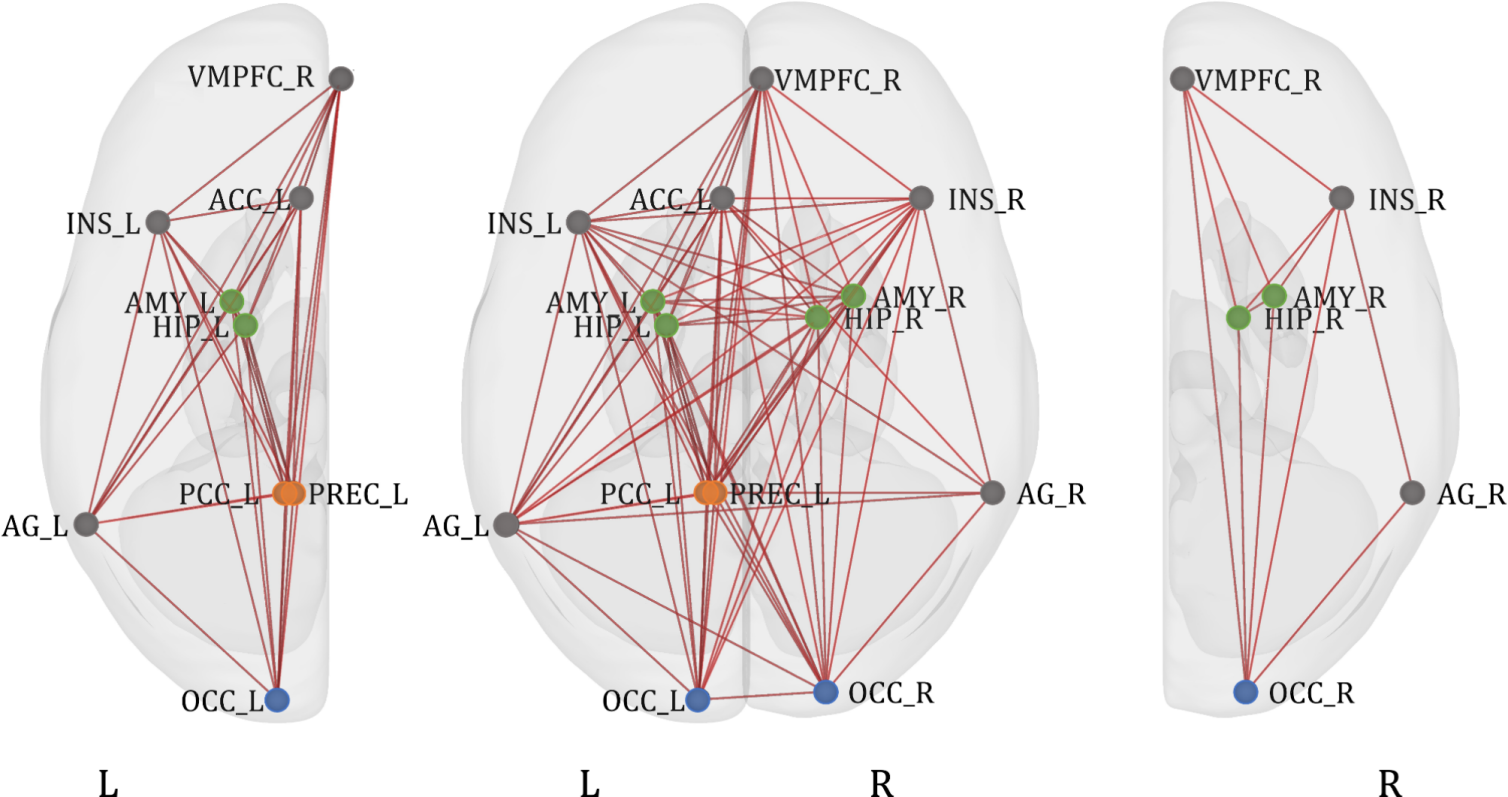
ROI-to-ROI functional connectivity of the main effect of task (SAD + HAPPY). Colors represent clusters of ROIs revealed in the hierarchical clustering procedure: orange - Cluster A, green - Cluster B, grey - cluster C, and blue - Cluster D (see Table 3 for the description of the clusters). The results were FDR-corrected at *p* < 0.05 for the cluster-level threshold (two-sided) together with an uncorrected *p* < 0.05 connection-level threshold for comparisons between individual connections. VMPFC - ventromedial prefrontal cortex; INS - insular cortex; ACC - anterior cingulate cortex; AMY - amygdala; HIP - hippocampus; AG - angular gyrus; PCC - posterior cingulate cortex; PREC - precuneus; OCC - occipital cortex.

The analysis of the main effect of task (SAD + HAPPY) revealed strong significant connections between and within all the clusters (Fig. 4; detailed table with results can be found in supplementary materials S1).

**Fig. 5:**
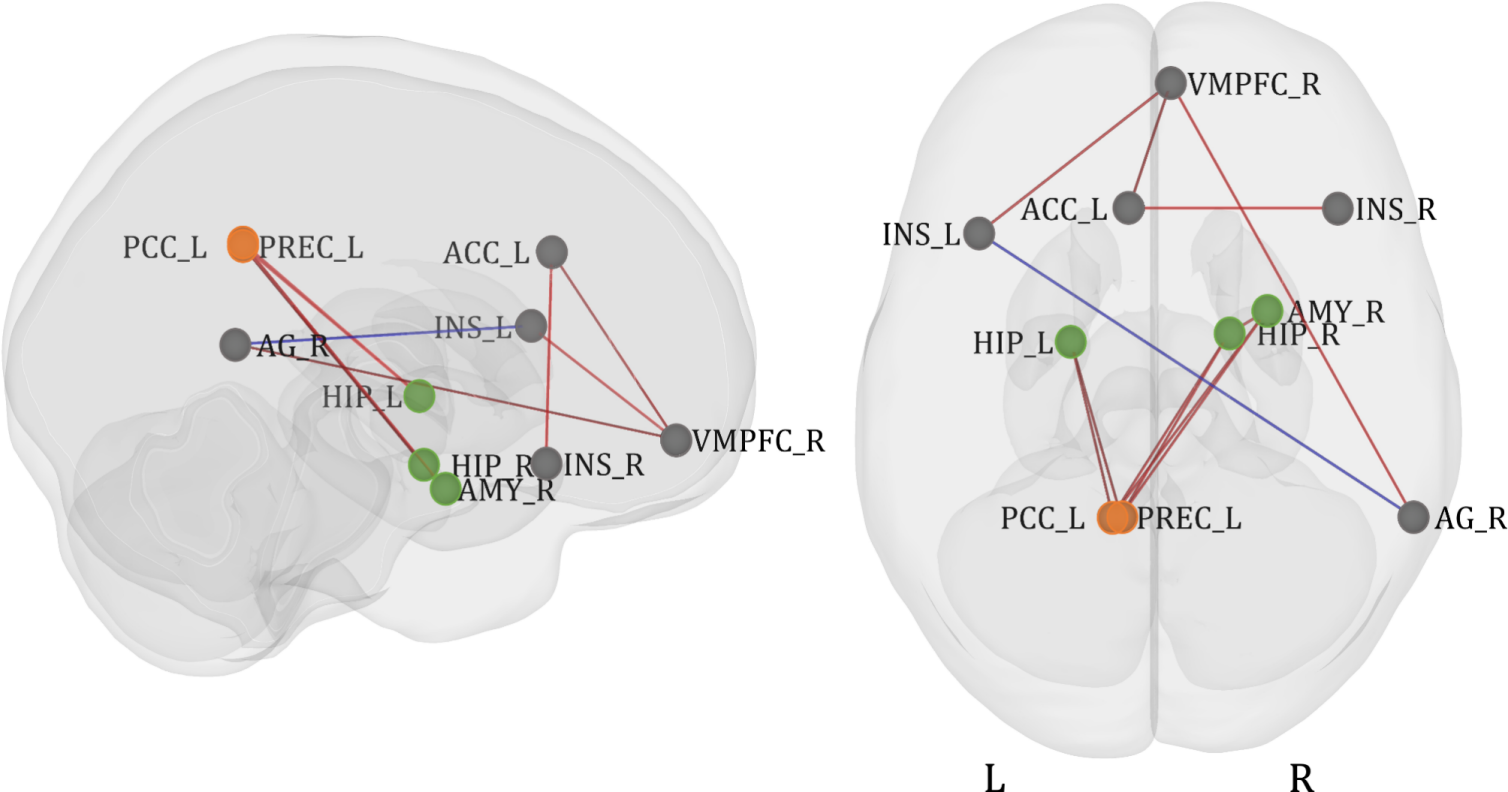
ROI-to-ROI functional connectivity of the comparison of sad and happy AMs recall (SAD > HAPPY) for all participants. The red color of lines indicates increased connectivity, whereas the blue color indicates decreased connectivity. Orange color represents ROIs from Cluster A, green color represents Cluster B, and grey color represents Cluster C. The results were FDR-corrected at *p* < 0.05 for the cluster-level threshold (two-sided) together with an uncorrected *p* < 0.05 connection-level threshold for comparisons between individual connections. L - left hemisphere; R - right hemisphere; PCC - posterior cingulate cortex; PREC - precuneus; ACC - anterior cingulate cortex; AG - angular gyrus; INS - insular cortex; HIP - hippocampus; VMPFC - ventromedial prefrontal cortex; AMY - amygdala.

An analysis of possible differences between sad and happy AMs recall for all participants taken together revealed 3 groups of connections that had significantly greater functional connectivity during sad AMs (SAD > HAPPY; Table 4; Fig. 5). There were no clusters with significantly increased connectivity for happy memories.

**Table 4:** Functional connectivity results of the comparison of sad and happy AMs recall (SAD > HAPPY) for all participants.

Clusters and connections	Statistic (df)	*p* FDR-corrected
***SAD > HAPPY***		
*Within Cluster C*	*F*(3, 79) = 4.40	**0.03**
Insula L - vmPFC R	*T* = 3.31	**0.02**
AG R - vmPFC R	*T* = 2.47	0.08
AG R - Insula L	*T* = -2.42	0.12
Insula R - ACC L	*T* = 2.36	0.19
ACC L - vmPFC R	*T* = 2.10	0.19
*Within Cluster B*	*F*(3, 79) = 4.31	**0.03**
Amygdala R - Hippocampus R	*T* = 3.62	**0.01**
*Between Clusters A and B*	*F*(3, 79) = 4.26	**0.03**
Precuneus L - Amygdala R	*T* = 2.79	**0.04**
Precuneus L - Hippocampus R	*T* = 2.71	**0.04**
Precuneus L - Hippocampus L	*T* = 2.66	**0.04**
PCC L - Hippocampus R	*T* = 2.66	0.1
PCC - Hippocampus L	*T* = 2.43	0.1
PCC L - Amygdala R	*T* = 2.31	0.1

Significant *p* values are written in bold. The analysis was FDR-corrected at *p* < 0.05 for the cluster-level threshold (two-sided) together with an uncorrected *p* < 0.05 connection-level threshold for comparisons between individual connections. L - left hemisphere; R - right hemisphere; vmPFC - ventromedial prefrontal cortex; AG - angular gyrus; ACC - anterior cingulate cortex; PCC - posterior cingulate cortex.

**Fig. 6:**
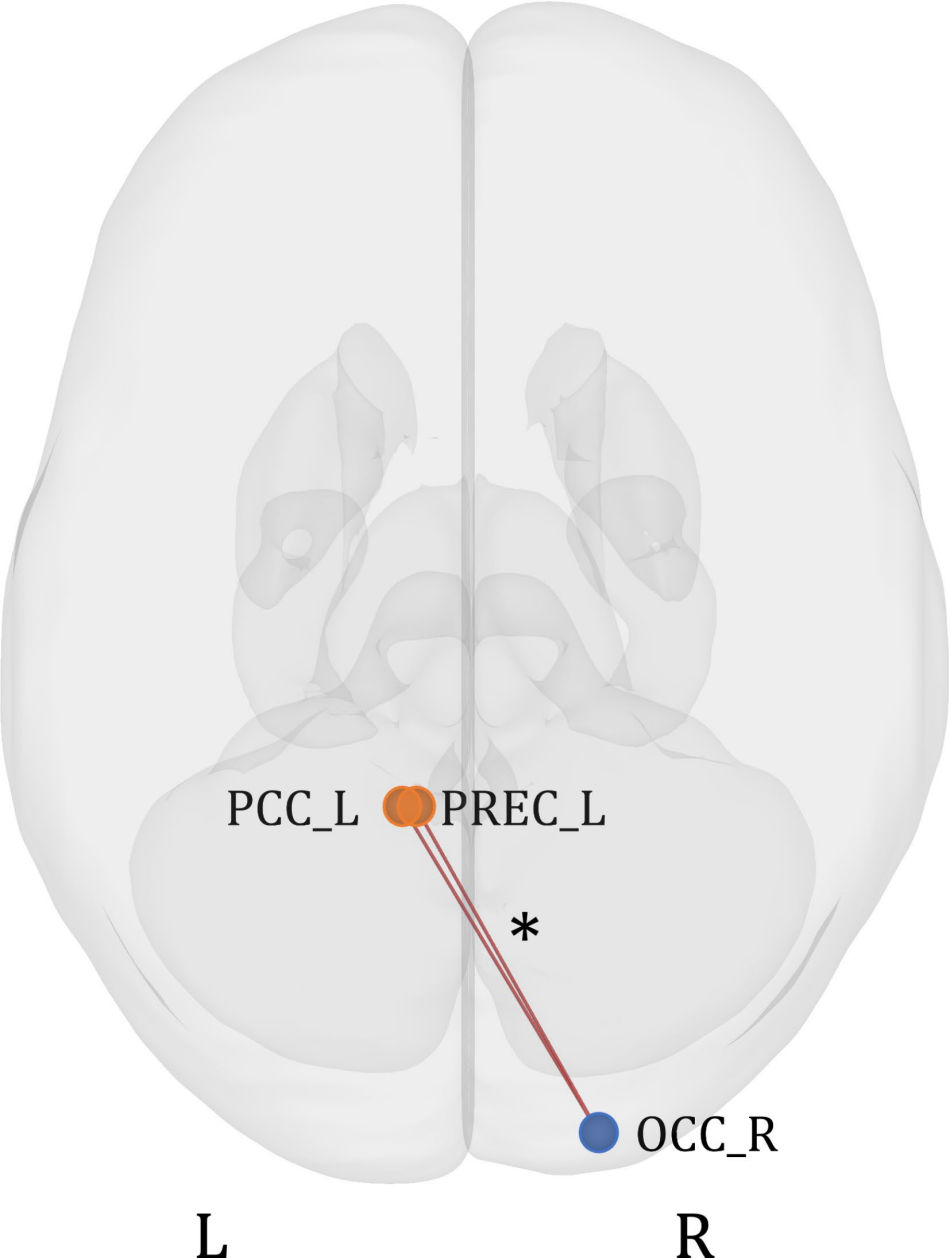
ROI-to-ROI functional connectivity of the comparison of clinical groups taken together to the healthy control group (MDD + BPD > HC). The orange color represents ROIs from Cluster A, and the blue color represents Cluster D. The results were FDR-corrected at *p* < 0.05 for the cluster-level threshold (two-sided) together with an uncorrected *p* < 0.05 connection-level threshold for comparisons between individual connections. L - left hemisphere; R - right hemisphere; PCC - posterior parietal cortex; PREC - precuneus; OCC - occipital cortex. *p < 0.05.

The main effect of the group for all AMs (SAD + HAPPY) revealed one significant group of two connections (*F*(4, 156) = 3.97, *p* uncorrected = 0.004, *p* FDR-corrected = 0.04). The connections were noted between Clusters A and D: between the left PCC and right occipital cortex (*F*(2, 79) = 4.75, *p* uncorrected = 0.01, *p* FDR-corrected = 0.1), and between the left precuneus and right occipital cortex (*F*(2, 79) = 5.61, *p* uncorrected = 0.01, *p* FDR-corrected = 0.07). Since the *F* test for the group of connections was significant we decided to explore this effect with post-hoc comparisons. Pairwise post-hoc comparisons were not significant but the group effect was driven by the difference between the HC group and the MDD and BPD groups taken together (*F*(2, 78) = 6.04, *p* uncorrected = 0.004, *p* FDR-corrected = 0.04), with the same connections (Fig. 6). The connectivity between the right occipital cortex and left precuneus was greater for the clinical groups (*T* = 3.12, *p* uncorrected = 0.002, *p* FDR-corrected = 0.03). The other connection was still statistically insignificant (*T* = 2.83, *p* uncorrected = 0.01, *p* FDR-corrected = 0.08).

The group-by-condition interaction analysis did not show any significant results.

## Discussion

This study aimed to test the behavioral and neural processing of sad and happy memories in women with MDD, BPD, and healthy control. The literature on this topic is very limited, while knowledge about it could have important implications for therapeutic work. The sole studies that previously examined AM recall across both disorders focused on comparing self-reported memories concerning their specificity^31,32,47^. Additionally, in earlier studies on BPD, only negative memories, whether resolved or unresolved, were utilized^36,37^. Current research, as far as we know, is the first investigation of neural and behavioral processing underlying the recall of sad and happy memories in people with BPD and MDD.

Contrary to our expectations, there were no significant differences between the clinical groups in ratings of their emotional state during the task. Neither did we find differences between the MDD and control groups. However, the analyses revealed that the BPD group rated their emotional state as significantly sadder than the HC group. The memories were also evaluated by independent judges (supplementary material S3). The analyses of those ratings revealed that memories of the BPD group were generally less positive (i.e., received lower valence ratings) than memories of the HC group. This could suggest that women with BPD consistently encounter more negative events. This may be the case, as the development of this disorder is often associated with childhood trauma and a non-validating environment^44^, whereas major depression is not consistently connected to childhood hardships and may also be a response to adverse situations or extended stress in later life^48^. It might be that also women with BPD experience more negative events due to trait impulsivity and proneness to engaging in risky behaviors.

Assessments of vividness indicated that among all participants, sad AMs were perceived as less vivid than happy. This result was also found within the MDD and HC groups, but no difference was found in BPD participants. Lower vividness ratings of sad memories compared to happy ones aligns with findings from a study by Lindeman et al.^49^ in a group of healthy participants. The authors illustrated that positive memories were recalled more vividly than sad ones and that emotions related to positive AMs diminished less than those for negative memories. This phenomenon is referred to as a fading affect bias - negative emotions associated with negative AMs tend to decline more rapidly^50^. A lack of differences between the MDD and HC groups in the current study echoed previous findings^33,34,51^. This could suggest that memories’ sensory details are stored similarly in these groups and that vividness may not be a factor of AM disturbance in depression. No previous studies measured this variable in BPD groups, so we are unable to refer to the previous findings.

The main effect of AM recall from the neuroimaging data revealed activations within brain regions that have been previously identified as essential for this kind of memory: occipital cortex, middle temporal lobe regions, precentral and postcentral gyri, angular gyrus, ACC, insula, along with other areas^4–6,14,46,52^. When the sad and happy memories were contrasted across all participants collectively, significantly stronger brain activations were detected only for sad AMs in areas of the left mPFC and dmPFC, angular gyrus, PCC, and right insular cortex. Both the medial prefrontal cortex and PCC are often seen as vital for handling self-related information during AM recall^10,14,46^. One potential explanation is that sad memories encompass more self-related information. As the insular cortex is tied to processing bodily states and body awareness^53^, sad AMs might elicit greater bodily reactions during recall. But, since there was also greater activation of TPJ, which participates in mental imagery of one’s body^54^ and in perspective shifts during imagery^55^, sad memories might also correspond to more visualizations of oneself. Since the vividness ratings were lower for sad AMs, this activation could also hint at a more substantial effort invested in visual imagery during the recall. Furthermore, increased activation of the angular gyrus implies that sad AMs require more semantic knowledge processing^56^ or more effort for successful recollection^57^. The obtained results are different from some of the previous studies (which, notably, compared different types of memories only in healthy groups). For example, Piefke et al.^16^ compared positive and negative memories in healthy participants and reported that negative memories activated the right middle temporal gyrus. Markowitsch et al.^18^ and Pelletier et al.^19^ compared happy memories to sad ones in healthy participants and noted that sad memories led to heightened activity in the lateral orbitofrontal cortex and ventrolateral prefrontal cortex. Additionally, Markowitsch et al.^18^ reported increased activity in the lateral temporal cortex, while Pelletier et al.^19^ noted increased activity in the pons. It’s worth mentioning that Vandekerckhove et al.^15^ did not find significant differences in brain activity when comparing negative, stressful, positive, and neutral memories in healthy participants. The absence of significantly stronger activations for happy AMs in our research contrasts with previous studies that indicated a higher engagement of areas such as OFC, mPFC, precuneus, or temporal cortex^18,19,58,59^. However, those studies included only healthy participants, whereas we compared the memories across all groups taken together. The lack of significant results for happy AMs might imply that they were less captivating or easier to recall even though there were no significant differences between the groups (which is described in the further part of the discussion).

Even though the study showed the effectiveness of the AM recall task, expected between-group differences in brain activations were not found. Our study did not replicate previously reported differences between groups with MDD and HC. Previous studies showed, for example, lower activation in MDD in regions of the PFC or medial temporal gyrus during recall in general^21^, diminished activation of the parietal and limbic cortices, and higher activation of medial temporal gyrus and PFC for positive AMs^33–35^, or higher activation of the ACC, amygdala, and hippocampus for negative memories^33,35^. One of the possible explanations is that the studied groups were too small, specifically the BPD group (*N* = 18). However, the current literature on the topic is based on similar or smaller samples and obtained significant group differences (for example, 15–24 participants;^36,64,65^). At the start of the study, we initially recruited and assessed a larger number of participants (42 in MDD group and 32 in the BPD group). However, women taking medication were later excluded from the final analyses due to the heterogeneity in dosages and substances prescribed. To ensure that the lack of significant results was not due to insufficient statistical power, we conducted additional analyses including all participants, with and without medication status as a covariate. Neither the main effect of group nor the interaction between group and condition yielded significant results, suggesting that the sample size was not responsible for the absence of significant differences. Another possible reason could be that participants’ symptoms have been too mild since the studied women were high functioning. On the other hand, people with more severe symptoms are harder to recruit, and also an ethical question remains, whether participation wouldn’t be risky for them and aggravate their symptoms more. Those patients are also more often prescribed multiple different medications, which are difficult to be accounted for in statistical analyses. Considering that both disorders are heterogenous in clinical manifestations^66,67^, group differences may occur less frequently. It is also difficult to define how often the lack of differences occurs because of the positive-results bias.

Moreover, since some of the women with BPD met the criteria for a current depressive episode, perhaps the clinical groups were too similar, and that impeded finding significant between-group differences. Some of the symptoms and mechanisms could have overlapped. However, BPD often co-occurs with depressive episodes in the general population and such recruitment of the study group may better reflect reality. Moreover, the analysis of covariance with occurrence of depressive episode as a covariate did not yield significant results in this study. Therefore, possible other variables influenced the lack of results and not diagnosis of depression. Lastly, there is a possibility of a lack of differences between these groups arising from shared neuropsychological profiles^68^. Functional connectivity analysis supported the current literature’s claims that such regions as vmPFC, amygdala, ACC, hippocampus, PCC, or angular gyrus, among others, are the most crucial for AM recall^4–6^ as we found significantly heightened connectivity between all regions of interest during recall of all AMs (sad and happy ones). Further analysis showed that only sad memories resulted in greater positive connectivity between multiple ROIs. Enhanced connectivity of the vmPFC with the insula and AG implies that sad memories lean heavily on integrating self-relevant details (vmPFC), processing emotional and physical cues (insula), and semantic recall (AG)^56,57^. Notably, sad AM recall showed increased connectivity between the amygdala and hippocampus, a result observed in general AM recall in past studies, suggesting that heightened emotions amplify memory search and retrieval [e.g.,^11,12^]. Therefore, sad AMs may be more dependent on this mechanism. There was also heightened connectivity between the precuneus, amygdala, and hippocampi during sad memory recall. Given the reduced vividness ratings for these memories, this suggests that these connections possibly support the successful recall of faded memories, integrating visual imagery and emotional significance^60,61^.

Further analysis revealed significant group differences between the combined clinical groups and the HC group. This demonstrated a stronger positive correlation between the left precuneus and right occipital cortex in MDD and BPD groups. It can suggest that vivid AM recall in these disorders requires better cooperation between visual imagery areas (occipital cortex) and contextual recall areas (precuneus). The precuneus might also link imagery to a more profound self-processing in clinical groups. Earlier research has suggested the precuneus plays a role in the third-person perspective during AM recall^62^ and becomes more active during distancing oneself from negative stimuli^63^. The clinical groups might use this perspective to distance themselves from emotional memories more often. Yet, we didn’t account for the memory perspective, and the precuneus activity didn’t differ across groups. Given this unique finding, further study is warranted.

This study investigated the behavioral and neural processing of sad and happy autobiographical memories in women with MDD, BPD, and healthy controls. Contrary to expectations, no significant differences were found between MDD and BPD groups in emotional state ratings or brain activations during memory recall. If the absence of group differences points to MDD and BPD sharing similar cognitive and emotional processing mechanisms when recalling autobiographical memories, it implies that interventions targeting autobiographical memory distortions or emotional responses to personal memories might be similarly effective across both disorders. The lack of differences also supports the utility of transdiagnostic approaches, which focus on treating underlying cognitive and emotional processes which can be unnoticed when focusing on categorical diagnosis. However, the neural data showed increased connectivity between regions involved in visual imagery and contextual memory in clinical groups, suggesting that these disorders may share underlying cognitive and emotional processes during memory recall. This novel result may suggest a need for stronger coordination between visual imagery and contextual recall for vivid memory retrieval in these clinical groups. Future research should aim to confirm and further explore this result.

## Limitations of the study and future directions

The study has some limitations. As mentioned in the previous section, the group sizes, were medium (MDD, HC) to small (for BPD). However, recruiting larger samples of women with depression or BPD is challenging due to co-occurring conditions, prescribed medications, and higher drop-out rates. Yet, our sample sizes align with previous studies. The memory collection method poses potential issues as the pre-scan interview executed a few days before the fMRI scan, might have prompted memory rehearsal. Additionally, the memories weren’t categorized by their remoteness, and while some research indicates that it affects the neural processing of AMs [e.g.,^69^], others find no such influence on such areas as the hippocampus^70,71^. The block design employed in the study could have potentially led to habituation to the emotional content of the blocks, although the emotional memories were interleaved with neutral ones. Future studies could employ more randomized sequences of memories. Lastly, excluding male participants narrows the study’s generalizability. However, a focus on exclusively women was established based on a higher diagnosis rate of both disorders in women and our and based on our experience in research studies, men are less likely to participate. Focusing on female participants may be regarded, in fact, as a strength of this research as it decreases the heterogeneity of our sample.

Future studies should aim to address some of the limitations in current research. One critical step forward is the recruitment of larger and more diverse participant groups. Increasing sample sizes would not only enhance the statistical power of studies but also allow for more nuanced subgroup analyses, leading to more generalizable findings. This includes recruitment of equal numbers of men and women. Ensuring gender balance in study samples, although challenging, could help to uncover potential gender differences in symptomatology and treatment responses. Additionally, future studies should aim to include a group of women with BPD who do not have co-occurring depression. This would provide clearer insights into the specific neural and cognitive characteristics of BPD. Moreover, describing symptomatology as a continuous variable rather than categorically could yield more nuanced insights into how symptoms vary along a spectrum, rather than treating them as all-or-nothing phenomena. This dimensional approach for both depressive and BPD symptoms would allow for the exploration of the full range of symptom severity and its impact on neural and cognitive functioning, offering a more detailed picture of the underlying pathology. Regarding the task design, future research could also focus on controlling from which perspective do the participants recall their experiences, as this would be important for more detailed conclusions.

## Methods

### Ethics declarations

The study was approved by the ethics committee at the Faculty of Psychology, University of Warsaw (approval: June 31st, 2016), and by the Ethics and Bioethics Committee at the Cardinal Stefan Wyszyński University in Warsaw (identification number: KEiB-09/2020). The study was conducted in accordance with the Code of Ethics of the World Medical Association (Declaration of Helsinki). All participants gave written informed consent and were paid for their participation in the study.

### Participants

A total of 82 participants were studied: 30 in the MDD group, 18 in the BPD group, and 34 in the healthy control group (HC). Study groups consisted only of women. The participants were matched in terms of age and years of education. The groups were not significantly different from each other neither in terms of age (*F*(2, 79) = 0.69, *p* = 0.5) nor of years of education (*F*(2, 79) = 2.68, *p* = 0.07). None of the MDD participants had comorbid borderline personality disorder. 11 women in the BPD group had a co-occurring depressive episode. All participants were unmedicated.

Participants were recruited through social media postings. The postings were separate for MDD and BPD groups, with each focusing on the symptoms of each disorder. Additionally, independent postings were directed at a healthy control group. Participants first filled out an online recruitment form, consisting of questions regarding the history of mental and neurological disorders, medication intake, psychotherapy experience, metal objects in the body, pregnancy, and claustrophobia, which were based on the inclusion and exclusion criteria. Based on the results of the recruitment form, respondents were scheduled for a psychological interview, conducted by a trained clinician, consisting of the MINI-International Neuropsychiatric Interview (MINI, V. 5.0.0;^72^) and the Structured Clinical Interview for DSM-5 Personality Disorders (SCID-5-PD;^73^). Inclusion criteria included: female sex, right-handedness, age between 18 and 50 years, and proficiency in the Polish language. Additional criteria for clinical groups were a current depressive episode for the MDD group and BPD diagnosis for BPD group. Exclusion criteria included: a history of neurological disorders or brain injuries, current intake of psychiatric medication, any conditions preventing participation in an MRI study, a history of psychotic, bipolar, or eating disorders, current alcohol or drug dependence, and strong suicidal ideations. Additional exclusion criteria for clinical groups were co-occurring personality disorders in the MDD group, co-occurring antisocial or schizotypal personality disorders in BPD group. The demographic and clinical characteristics of the groups are presented in Table 5.

**Table 5:** Demographic and clinical characteristics of the participants. MDD – major depressive disorder, BPD – borderline personality disorder, HC – healthy control, SD – standard deviation.

	MDD (*N* = 30)			HC (*N* = 34)		
	Mean	SD	Range	Mean	SD	Range
*Demographic data*						
Age	28.2	6.76	21–47	28.09	6.31	19–44
Years of education	17.3	2.25	12–22	17.13	2.95	11–24
	*n*	*%*				
*Comorbid psychiatric disorders*						
Depressive episode	-	-				
Dysthymia	8	26.67				
Panic attacks	4	13.33				
Agoraphobia	1	3.33				
Social phobia	2	6.67				
Obsessive-compulsive disorder	0	0				
Post-traumatic stress disorder	0	0				
General anxiety disorder	0	0				
*Comorbid personality disorders*						
Dependent	-	-				
Avoidant	-	-				
Paranoid	-	-				
Schizoid	-	-				
Obsessive-compulsive	-	-				
Narcissistic	-	-				

### Experimental procedure

One to two weeks before the fMRI scan, all study participants met with one of two study authors (MK or KR) in person or online and were asked to provide 5 sad (SAD) and 5 happy (HAPPY) memories, and 5 neutral daily situations (NEUTRAL) which elicited no emotions. The recall of neutral memories was designed in line with the previous studies [e.g.,^34^], and was used as a break from emotional information. Neutral memories were not treated as a control condition in the fMRI analyses. Each memory was shortened to a cue and was implemented in the fMRI task to trigger active recall (see the supplementary material S2 for examples of memories and cues). Prior to the MRI scan, participants were told that while a cue was presented on the screen they should try and recall as many details of that corresponding memory as they could, including circumstances, surroundings, places, people, and emotions. Participants were also trained on how to answer behavioral questions during the scan with a response pad.

**Fig. 7:**
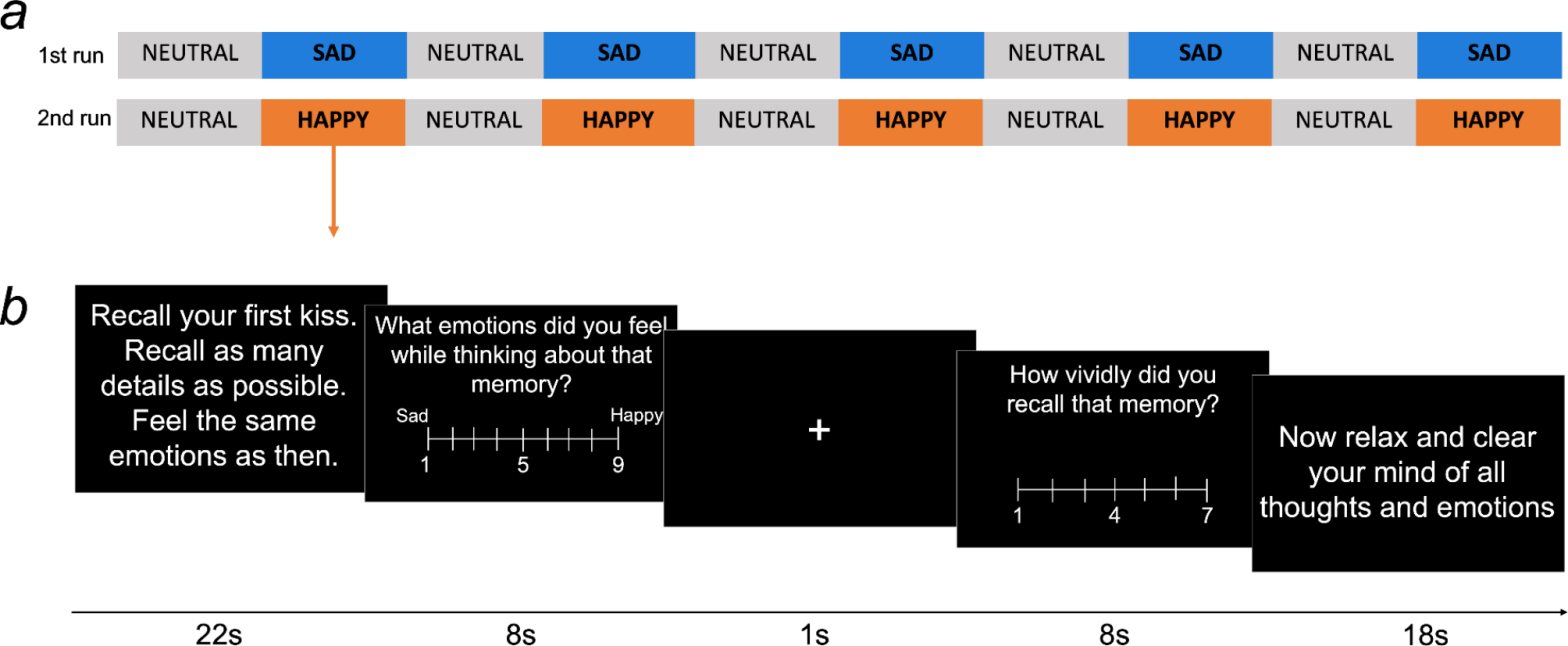
The experimental design of the task. (**a**) Structure of the functional runs. Each run consisted of 10 trials and lasted for around 10 min. The same five neutral situations were repeated in both runs but their order was randomized. (**b**) Structure of a trial. Each started with a cue and an instruction. After the recall, participants answered two questions about their affective state and the vividness of a memory. NEUTRAL - the recall of neutral memories, SAD - the recall of sad memories, HAPPY - the recall of happy memories.

The scanning session for this task included 2 functional runs: a run with sad memories and neutral situations and a run with happy memories and neutral situations. Each run had 10 interleaved trials in a fixed order (see Fig. 7a), including 5 trials with emotional memories and 5 trials with neutral situations. The neutral events were the same for both runs but their order was randomized.

Each trial (see Fig. 7b) lasted 57 s and began with instruction and a cue and time for the recall (22s). The recall period was immediately followed by two questions (8s each), which were separated by a fixation cross (1s). The first question regarded emotions during recall (“*What emotions did you feel while thinking about that memory?*”) and participants answered on a 9-point Likert scale (where 1 indicated strong sadness, 5 indicated a neutral state, and 9 indicated strong happiness). The second question asked about the vividness of the memory (“*How vividly did you recall the memory?*”) and had a 7-point Likert scale (where 1 indicated “not vivid at all, I could not remember anything” and 7 indicated “extremely vivid, I could remember everything”). Trials ended with a relaxation period (18s). After this part ended, the next trial started. The experimental procedure was designed using Presentation software (Neurobehavioral Systems, http://www.neurobs.com/).

### MRI data acquisition

MRI data were acquired using a 3T Siemens Magnetom Trio scanner (Siemens Medical Solutions) equipped with a 32-channel head coil. The following images were acquired during a single scanning session: a structural localizer image, field map magnitude image (TR = 488ms, TE = 7.46ms, flip angle = 60°, voxel size = 3 × 3 × 2.5 mm, field of view = 216 mm), field map phase image (TR = 488ms, TE = 5ms, flip angle = 60°, voxel size = 3 × 3 × 2.5 mm, field of view = 216 mm), 2 series of functional EPI images (45 slices, slice thickness = 2.5 mm, TR = 2500ms, TE = 30ms, flip angle = 90°, field of view = 216 mm, voxel size = 3 × 3 × 2.5 mm), structural T1-weighted image (176 slices, slice thickness = 1 mm, TR = 2530ms, TE = 3.32ms, flip angle = 7°, field of view = 256 mm, voxel size = 1 × 1 × 1 mm).

fMRI data preprocessing.

The DICOM series were converted to NIfTI format using Horos Bids Output Extension (https://github.com/mslw/horos-bids-output). Preprocessing was performed with the Statistical Parametric Mapping program (SPM12, http://www.fil.ion.ucl.ac.uk/spm). Functional images were preprocessed using standard steps (Poldrack et al., 2011): correction for distortions related to magnetic field inhomogeneity using fieldmap images, correction for motion using realignment to the first acquired image, correction for differences between acquired slices, coregistration of the anatomical image to the mean functional image, normalization to the MNI space with 2 × 2 × 2 mm voxels and smoothing with 6 mm FWHM Gaussian kernel. To identify additional sources of movement artifacts in the functional images, the Artifact Detection Toolbox (ART, https://www.nitrc.org/projects/artifact_detect) was used, with a translation threshold of 2 mm and a rotation threshold of 0.04 radians. Images with motion exceeding these thresholds were considered outliers and were regressed out in the 1st level models.

### Behavioral analysis

Two 3 × 1 analyses of variance (ANOVA) models were used to check for differences between the groups in age and years of education.

An aligned rank transform for nonparametric repeated measures ANOVA^74,75^ was used in a 3 × 2 model, with a group (MDD, BPD, HC) as a between-subject variable, and condition (SAD, HAPPY) as a within-subject variable. Two separate models were used for each of the behavioral questions - a question about the emotional state during recall and a question about the vividness of memory. Post hoc tests were corrected using Holm’s correction for multiple comparisons. The analyses were performed in R Studio [^76^, http://www.rstudio.com/], with the use of ARTool^75,77^ and *emmeans*^78^ packages. All the sad and happy memories were rated by independent judges to investigate if any group had objectively sadder or happier memories (See supplementary material S3 for more information and results).

### fMRI data analysis

At the first-level analysis general linear modeling (GLM) was used to model the blood-oxygen-level-dependent signal (BOLD) for each participant. For each subject, the GLM consisted of 2 scanning sessions, each containing 1 trial regressor of interest: sad or happy memories (depending on the session). Neutral memories, relax after sad/happy memories, relax after neutral memories, behavioral questions, and a fixation cross between them, parameters of head motion, and ART motion regressors were added to the model as regressors of no interest. All the regressors related to the task were convolved with a standard hemodynamic response function (HRF). As each trial (containing a memory, two behavioral questions with a fixation cross between them, and a relaxation period) lasted for 57s, the expected trial-related signal changes had a period of 114s. In order not to filter those possible changes out, the high-pass filter was set to 228s - a value of four lengths of a single trial.

### Whole-brain GLM analysis

To test the main effect of task (emotional AM recall), the average activity in sad and happy memories (SAD + HAPPY) was analyzed across all groups taken together, using a one-sample t-test. A paired t-test was used to identify regions activated specifically by sad or by happy AMs across all participants (SAD > HAPPY and HAPPY > SAD contrasts). To test the main effect of group, the groups were contrasted for the average effect of sad and happy AMs (SAD + HAPPY) using a one-way ANOVA model. To test the interaction between the group and emotional memories, a flexible factorial model was used with a group (MDD, BPD, HC) as the between-subject factor and a condition (SAD, HAPPY) as the within-subject factor. Moreover, the presence of a current depressive episode (yes or no; 1/0) was added as a covariate in the analyses of the main effect of group and the interaction between group and condition to test for the potential influence of depression in BPD participatns on the results. Results were thresholded at a voxel-wise height threshold of *p* < 0.001 (uncorrected) combined with a cluster-level extent-threshold of *p* < 0.05, corrected for multiple comparisons using the family-wise error (FWE) rate. All reported brain regions are labeled according to the automated anatomical labeling (AAL2) atlas applied in bspmview (https://www.bobspunt.com/bspmview).

### Regions of interest analysis

Regions of interest (ROIs) were specified a priori. They corresponded to the main brain regions implicated in AM recall based on previous meta-analyses and AM literature [e.g.,^4–6,45,46^]. These ROIs were: vmPFC, hippocampus, amygdala, occipital cortex, precuneus, posterior and anterior cingulate cortices, insular cortex, and angular gyrus. Anatomical masks of these regions were taken from the AAL2 atlas, while the vmPFC mask was taken from a Neurovault collection (https://neurovault.org/images/132836/). These masks were used with the main effect of task model (SAD + HAPPY) and coordinates of the strongest peak activations within the masks were taken as the centers of ROIs, which were constructed as the spheres of 12 mm radius centered on these coordinates. The following regions and their corresponding coordinates were included in the final set of ROIs: right vmPFC (x = 4, y = 54, z = -14), left hippocampus (x = -20, y = -8, z = -12), right hippocampus (x = 18, y = -6, z = -16), left occipital cortex (x = -12, y = -102, z = 4), right occipital cortex (x = 20, y = -100, z = 6), left precuneus (x = -1, y = -50, z = 34), left PCC (x = -8, y = -50, z = 30), left ACC (x = -6, y = 24, z = 30), left insular cortex (x = -42, y = 18, z = -2), right insular cortex (x = 44, y = 24, z = -6), left angular gyrus (x = -60, y = -58, z = 26), and right angular gyrus (x = 62, y = -50, z = 30).For the bilateral amygdala, the whole mask was used due to its small anatomical volume. These ROIs were used for the small-volume correction (SVC) analyses with a sphere of 12 mm radius centered on these coordinates. A flexible factorial model was used to test the interaction with a group as the between-subject factor and condition as the within-subject factor.

### Functional connectivity analysis

The CONN toolbox^79^ for SPM was used to perform task-based functional connectivity analyses. First-level SPM files, including ART motion parameters, and normalized T-1 images were imported into the software. ROIs defined as spheres of 6 mm radius were centered on the same peak coordinates used for ROIs definition for GLM analysis. The 6 mm spheres were used as functional connectivity seeds. For the amygdalae two anatomical masks were used, taken from the AAL2 atlas. A denoising procedure in CONN was applied to data to remove confounding motion and physiological effects from the BOLD signal. Regressors for this procedure were: signals from white matter and cerebrospinal fluid, realignment parameters obtained from the SPM preprocessing, and ART movement covariates. Task effects were also included as regressors to avoid measuring connectivity caused by shared task-related co-activation responses between brain regions. The signal was high-pass filtered with 0.004 Hz which corresponds to a high-pass filter of 228s used in the GLM analysis.

Second-level analyses were performed using a weighted GLM approach with bivariate correlations. ROI-to-ROI correlations were computed among all the defined ROIs for each effect of interest. The ROIs were sorted automatically into clusters by a data-driven hierarchical clustering procedure called complete-linkage clustering^80^ based on ROIs anatomical proximity and functional similarity (connectivity patterns;^81^). This allows for the performance of analyses between and within clusters and reduces the number of comparisons. The Functional Network Connectivity (FNC) multivariate parametric statistics with default settings applied were used for cluster-level inferences. This approach allows for analysis of between-network connectivity for all the clusters and within-network connectivity for all connections within those clusters. All FNC analyses were corrected with a false discovery rate (FDR-corrected) at *p* < 0.05 for the cluster-level threshold (two-sided) together with an uncorrected *p* < 0.05 connection-level threshold for post hoc comparisons between individual connections.

To test the main effect of task (emotional AM recall), the average connectivity in sad and happy memories (SAD + HAPPY) was analyzed across all groups taken together, using a one-sample t-test. To test possible differences in functional connectivity between sad and happy AMs recall a two-tailed paired t-test was performed for all the subjects taken together. To test the main effect of group 3 × 1 ANOVA with the average effect of sad and happy AMs (SAD + HAPPY) was used. To test the interaction between group (MDD, BPD, HC) and condition (SAD, HAPPY) a 3 × 2 ANOVA model was used.

## Electronic supplementary material

Below is the link to the electronic supplementary material.

Supplementary Material 1

## Data Availability

Relevant data are stored in an OSF repository and are available at https://osf.io/k4dy5/. Unthresholded statistical maps from the reported comparisons are available at Neurovault, https://neurovault.org/collections/17134/.
