# Supplementary material for: Neural processing of sad and happy autobiographical memories in women with depression and borderline personality disorder
Source: Sci Rep. 2024 Dec 28;14:30884. doi: 10.1038/s41598-024-81840-x (PMC11681166; doi:10.1038/s41598-024-81840-x)
Supplement: Supplementary file 1 — Supplementary Material 1 [file 41598_2024_81840_MOESM1_ESM.pdf]

# Neural processing of sad and happy autobiographical memories in women with depression and borderline personality disorder

Maria Kulesza*[1], Katarzyna Rękawek[1,2], Paweł Holas[2], Dorota Żołnierczyk-Zreda[3], Marlena Sokół-Szawłowska[4], Anna Poleszczyk[5], Artur Marchewka[1], Marek Wypych*[1]

maria.k.kulesza@gmail.com
m.wypych@nencki.edu.pl

[1]Laboratory of Brain Imaging, Nencki Institute of Experimental Biology, Pasteura 3, 02-093, Warsaw, Poland
[2]Faculty of Psychology, University of Warsaw, Stawki 5/7, 00-183, Warsaw, Poland
[3]Laboratory of Psychology and Sociology, Central Institute for Labour Protection – National Research Institute, Czerniakowska 16, 00-701, Warsaw, Poland
[4]Outpatient Clinic, Institute of Psychiatry and Neurology, Sobieskiego 9, 02-957, Warsaw, Poland
[5]Department of Clinical Neurophysiology, Institute of Psychiatry and Neurology, Sobieskiego 9, 02-957, Warsaw, Poland

Supplementary information for „Neural processing of sad and happy memories in women with depression and borderline personality Disorder"

## S1. Functional connectivity of the main effect of task.

**Table S1.** The results were FDR-corrected at p < 0.05 for the cluster-level threshold (two-sided) together with an uncorrected p < 0.05 connection-level threshold for comparisons between individual connections. PCC - posterior cingulate cortex; OCC - occipital cortex; ACC - anterior cingulate cortex; AG - angular gyrus; vmPFC - ventromedial prefrontal cortex.

| Clusters and connections | Statistic | *p* FDR-corrected |
|---|---|---|
| ***Main effect of recall (SAD+HAPPY)*** | | |
| *Within Cluster A* | $F(1, 81) = 7594.41$ | < 0.001 |
| PCC L - Precuneus L | $T = 87.15$ | < 0.001 |
| *Within Cluster D* | $F(1, 81) = 342.09$ | < 0.001 |
| OCC L - OCC R | $T = 18.50$ | < 0.001 |
| *Within Cluster B* | $F(3, 79) = 308.75$ | < 0.001 |
| Amygdala R - Hippocampus R | $T = 27.35$ | < 0.001 |
| Amygdala L - Hippocampus L | $T = 27.11$ | < 0.001 |
| Amygdala L - Amygdala R | $T = 18.85$ | < 0.001 |
| Amygdala L - Hippocampus R | $T = 18.67$ | < 0.001 |
| Hippocampus L - Hippocampus R | $T = 14.94$ | < 0.001 |

| Clusters and connections | Statistic | *p* FDR-corrected |
|---|---|---|
| Hippocampus L - Amygdala R | $T = 13.18$ | < 0.001 |
| | | |
| *Within Cluster C* | $F(3, 79) = 148.92$ | < 0.001 |
| Insula L - Insula R | $T = 16.52$ | < 0.001 |
| Insula L - ACC L | $T = 16.06$ | < 0.001 |
| ACC L - Insula R | $T = 11.95$ | < 0.001 |
| AG L - AG R | $T = 11.17$ | < 0.001 |
| AG L - Insula R | $T = 7.64$ | < 0.001 |
| Insula L - AG L | $T = 7.13$ | < 0.001 |
| ACC L - AG R | $T = 6.99$ | < 0.001 |
| ACC L - AG L | $T = 6.64$ | < 0.001 |
| Insula L - AG R | $T = 5.96$ | < 0.001 |
| Insula R - vmPFC R | $T = 5.75$ | < 0.001 |
| Insula R - AG R | $T = 5.66$ | < 0.001 |
| AG L - vmPFC R | $T = 4.58$ | < 0.001 |
| ACC L - vmPFC R | $T = 3.88$ | < 0.001 |
| Insula L - vmPFC R | $T = 2.81$ | 0.01 |
| | | |
| *Between Clusters A and C* | $F(3, 79) = 104.93$ | < 0.001 |
| PCC L - vmPFC R | $T = 14.86$ | < 0.001 |
| Precuneus L - vmPFC R | $T = 13.31$ | < 0.001 |
| Precuneus L - AG L | $T = 11.28$ | < 0.001 |
| PCC L - AG L | $T = 11.25$ | < 0.001 |
| Precuneus L - AG R | $T = 7.53$ | < 0.001 |
| PCC L - AG R | $T = 7.11$ | < 0.001 |
| PCC L - Insula R | $T = 6.46$ | < 0.001 |
| Precuneus L - Insula R | $T = 6.03$ | < 0.001 |
| PCC L - Insula L | $T = 4.74$ | < 0.001 |
| PCC L - ACC L | $T = 4.72$ | < 0.001 |
| Precuneus L - Insula L | $T = 4.35$ | < 0.001 |
| Precuneus L - ACC L | $T = 4.29$ | < 0.001 |
| | | |
| *Between Clusters B and C* | $F(3, 79) = 72.24$ | < 0.001 |
| Amygdala L - vmPFC R | $T = 9.95$ | < 0.001 |
| Hippocampus R - vmPFC R | $T = 9.87$ | < 0.001 |
| Hippocampus L - vmPFC R | $T = 9.41$ | < 0.001 |
| Amygdala R - Insula R | $T = 8.26$ | < 0.001 |
| Amygdala L - ACC L | $T = 8.04$ | < 0.001 |
| Amygdala L - Insula R | $T = 7.02$ | < 0.001 |
| Amygdala R - vmPFC R | $T = 6.78$ | < 0.001 |
| Amygdala L - Insula L | $T = 6.52$ | < 0.001 |
| Hippocampus L - AG L | $T = 6.39$ | < 0.001 |

| Clusters and connections | Statistic | *p* FDR-corrected |
|---|---|---|
| Hippocampus R - Insula R | *T* = 6.35 | < 0.001 |
| Amygdala R - ACC L | *T* = 6.02 | < 0.001 |
| Hippocampus L - Insula R | *T* = 5.72 | < 0.001 |
| Hippocampus R - AG L | *T* = 4.75 | < 0.001 |
| Amygdala L - AG L | *T* = 4.69 | < 0.001 |
| Amygdala R - Insula L | *T* = 4.25 | < 0.001 |
| Amygdala R - AG L | *T* = 4.08 | < 0.001 |
| Hippocampus L - Insula L | *T* = 4.06 | < 0.001 |
| Hippocampus R - ACC L | *T* = 3.75 | < 0.001 |
| Hippocampus R - Insula L | *T* = 3.11 | 0.003 |
| Hippocampus L - ACC L | *T* = 3.05 | 0.003 |
| *Between Clusters A and D* | F(2, 80) = 36.96 | < 0.001 |
| PCC L - OCC R | *T* = 8.52 | < 0.001 |
| Precuneus L - OCC R | *T* = 8.18 | < 0.001 |
| PCC L - OCC L | *T* = 6.56 | < 0.001 |
| Precuneus L - OCC L | *T* = 6.32 | < 0.001 |
| *Between Clusters A and B* | *F*(3, 79) = 34.18 | < 0.001 |
| Precuneus L - Hippocampus R | *T* = 1.13 | < 0.001 |
| PCC L - Hippocampus R | *T* = 1.06 | < 0.001 |
| PCC L - Hippocampus L | *T* = 9.21 | < 0.001 |
| Precuneus L - Hippocampus L | *T* = 8.62 | < 0.001 |
| PCC L - Amygdala L | *T* = 6.95 | < 0.001 |
| Precuneus L - Amygdala L | *T* = 6.85 | < 0.001 |
| Precuneus L - Amygdala R | *T* = 5.08 | < 0.001 |
| PCC L - Amygdala R | *T* = 5 | < 0.001 |
| *Between Clusters D and C* | *F*(3, 79) = 33.05 | < 0.001 |
| OCC R - vmPFC R | *T* = 9.33 | < 0.001 |
| OCC L - Insula R | *T* = 6.39 | < 0.001 |
| OCC L - vmPFC R | *T* = 5.77 | < 0.001 |
| OCC R - AG L | *T* = 5.72 | < 0.001 |
| OCC L - AG L | *T* = 5.20 | < 0.001 |
| OCC R - Insula R | *T* = 4.91 | < 0.001 |
| OCC L - Insula L | *T* = 4.41 | < 0.001 |
| OCC R - Insula L | *T* = 4.35 | < 0.001 |
| OCC L - ACC L | *T* = 3.40 | 0.001 |
| OCC R - ACC L | *T* = 2.92 | 0.006 |
| OCC R - AG R | *T* = 2.34 | 0.02 |
| *Between Clusters D and B* | *F*(3, 79) = 9.71 | < 0.001 |
| OCC L - Amygdala L | *T* = 4.74 | < 0.001 |

| Clusters and connections | Statistic | $p$ FDR-corrected |
|---|---|---|
| OCC R - Hippocampus L | $T = 4.20$ | < 0.001 |
| OCC L - Hippocampus R | $T = 3.84$ | < 0.001 |
| OCC L - Hippocampus L | $T = 3.35$ | 0.001 |
| OCC R - Amygdala L | $T = 3.35$ | 0.002 |
| OCC R - Hippocampus R | $T = 2.77$ | 0.01 |
| OCC L - Amygdala R | $T = 2.43$ | 0.02 |
| OCC R - Amygdala R | $T = 2.03$ | 0.05 |

**S2. The examples of memories provided by three participants.**

Below are examples of one sad memory, one happy memory, and one neutral situation for three different participants, and the cues that appeared during the task.

**Table S2**. Examples of memories and memory cues from three participants.

| | **Memory/cue type** | **Content of the memory** |
|---|---|---|
| **Participant 1** | Sad memory | When my fiancé forgot about our first anniversary and spent it with his friends, it still „sits" in me very hard. |
| | Sad memory cue | Recall *forgetting about the first anniversary.* |
| | Happy memory | I picked up my current cat from [city] and it was my first contact with a bald cat. |
| | Happy memory cue | Recall *picking up a cat from* [city]. |
| | Neutral situation | Brushing teeth. |

| | Memory/cue type | Content of the memory |
|---|---|---|
| | Neutral situation cue | Recall the last time you *brushed your teeth.* |
| **Participant 2** | Sad memory | When my boyfriend after 3,5 years told me that he doesn't know if he loves me and broke up with me. |
| | Sad memory cue | Recall *the breakup after 3,5 years.* |
| | Happy memory | Swimming in a lake at night. This was my first sailing trip, we were sailing most of the day, then had a bonfire. When it was dark already, we were going to swim in a lake. It was a very starry night. |
| | Happy memory cue | Recall *swimming in a lake at night.* |
| | Neutral situation | Eating dinner. |
| | Neutral situation cue | Recall the last time you *were eating dinner*. |
| **Participant 3** | Sad memory | In April [year] my dad called me with the news that my grandmother had died. She was sick for a while, so it wasn't a big surprise, but it was very sad in itself. The next day we were supposed to go to her, and we didn't make it. |
| | Sad memory cue | Recall *your grandmother's death.* |
| | Happy memory | Passing my driving license test was a joyful moment. Unfortunately, I failed the 1st time, but not during the second time, and I was able to pass with a lady who was widely known to be rigorous. |
| | Happy memory cue | Recall *passing the driver's license test.* |
| | Neutral situation | Washing hair. |
| | Neutral situation cue | Recall the last time you *washed your hair.* |

## S3. Ratings of autobiographical memories by independent judges

S3.1. Methods

In this part of the study 9 women participated as independent judges. All were in their late twenties and had higher education. They were asked to rate only sad and happy memories - together 820 memories from all the participants. The AMs were randomly divided into three groups (A, B, and C) and each group was rated by 3 of the judges. The judges were informed that the memories were gathered from women over 18 years old as part of a doctoral project but were blinded to the fact that some of the participants had clinical diagnoses. They were instructed to think about what level of sadness/happiness, arousal, and valence these memories trigger in the person to whom they belong when this person recalls a given memory. Some aspects of the memories were anonymized to protect participants' identity, for example, a city's name was changed or erased. Each memory was rated on three scales:

1) sadness (for sad AMs) or happiness (for happy AMs) on a 7-point Likert scale, where 1 indicated lack of sadness (happiness), and 7 indicated the highest intensity of sadness (happiness),

2) valence on a 9-point Self-Assessment Manikin scale, where 1 meant that a memory elicited very negative emotions, and 9 meant that a memory elicited very positive emotions,

3) arousal on a 9-point Self-Assessment Manikin scale, where 1 meant that a memory elicited a sense of calmness, and 9 meant that a memory elicited very high emotional arousal.

In order to analyze the reliability of judges' ratings Intraclass Correlation Coefficients (ICC; Koo and Li, 2016) estimates and their 95% confidence intervals were calculated. The ICC were calculated for each group of judges separately using a mean-rating ($k = 3$), absolute-agreement, 2-way mixed-effects models.

Analysis was performed using 3 statistical models with aligned rank transform for nonparametric factorial ANOVA (Fawcett and Salter, 1984; Wobbrock et al., 2011). In order to compare participant groups on how sad the sad AMs were, a 3x1 model was used with a group (MDD, BPD, HC) as a between-subject variable, and with sad memories as a within-subject variable. The same comparison was performed for the happy AMs. To compare groups and memories on valence and arousal scales, two 3x2 models were used with group (MDD, BPD, HC) as a between-subject variable, and with condition (sad and happy memories) as a within-subject variable. Post hoc tests were corrected using Holm's correction for multiple comparisons. Described analysis was performed in R Studio (RStudio Team, 2019, http://www.rstudio.com/), with the use of *ARTool* (Wobbrock et al., 2011; Kay et al., 2021), *emmeans* (Lenth, 2019), and *irr* packages (Gamer et al., 2019).

S3.2. Results

The ICC analysis showed good reliability within all 3 groups of judges, which means that their answers were well related to each other (Table S4).

**Table S3**. Reliability estimates for independent judges' AMs ratings. ICC - Intraclass Correlation Coefficients; CI – confidence interval.

| Group of judges | ICC | 95% CI |
|---|---|---|
| A | 0.79 | 0.65 - 0.86 |
| B | 0.83 | 0.81 - 0.85 |
| C | 0.87 | 0.78 - 0.91 |

Concerning the analyses of judges' ratings, the analysis of sadness ratings did not reveal any significant results. The analysis of happiness ratings revealed a significant main effect of group ($F(2, 79) = 3.28$, $p = 0.04$, $\eta 2 = 0.08$). Post hoc comparisons were found to be statistically insignificant.

The analysis of valence ratings revealed a significant main effect of group ($F(2, 79) = 4.64$, $p < 0.05$, $\eta 2 = 0.11$) and of condition ($F(1, 79) = 399.69$, $p < 0.001$, $\eta 2 = 0.83$), but no significant effect of interaction between group and condition ($F(2, 79) = 0.65$, $p = 0.5$, $\eta 2 = 0.02$). Post hoc comparisons of the main effect of group showed significantly lower valence ratings of the BPD group's AMs than those of the HC group ($T = -2.78$, $p = 0.02$), but no significant differences between the valence ratings of MDD and HC groups' AMs ($T = 2.26$, $p = 0.054$). The comparison between BPD and MDD groups was not statistically significant ($T = -0.8$, $p = 0.43$). Post hoc comparison of the main effect of condition showed that happy AMs had significantly higher valence ratings than sad AMs ($T = 19.37$, $p < 0.001$) (Figure S2).

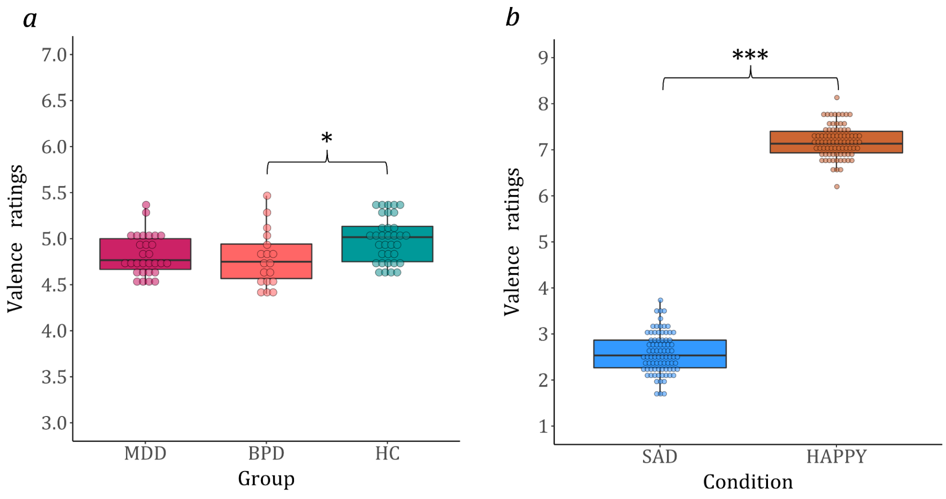


**Figure S1**. Behavioral results of judges' valence ratings. (a) Main effect of group. (b) Main effect of condition. Dots represent mean values for individual participants. The lower and upper

borders of the box correspond to the first and third quartiles, respectively. The lower and upper whiskers represent the smallest and largest data points, respectively, no further than 1.5 x interquartile range from the borders. $^*p < 0.05$, $^{***}p < 0.001$

The analysis of arousal ratings revealed a significant main effect of condition ($F(1, 79) = 135.38$, $p < 0.001$, $\eta 2 = 0.63$), but no significant main effect of group ($F(2, 79) = 1.99$, $p = 0.1$, $\eta 2 = 0.05$) and no significant interaction between group and condition ($F(2, 79) = 1.7$, $p = 0.2$, $\eta 2 = 0.04$). Post hoc comparison of the main effect of condition revealed significantly lower arousal ratings for happy AMs than for sad ones ($T = -11.2$, $p < 0.001$) (Figure S2).

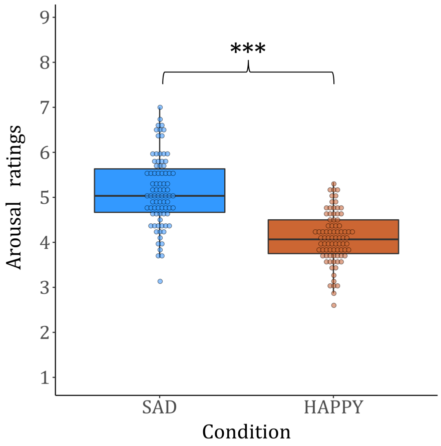


**Figure S2**. Behavioral results of judges' arousal ratings - the main effect of condition. Dots represent mean values for individual participants. The lower and upper borders of the box correspond to the first and third quartiles, respectively. The lower and upper whiskers represent the smallest and largest data points, respectively, no further than 1.5 x interquartile range from the borders. $^{***}p < 0.001$
